# 2109. *In vitro* and *in vivo* Killing Kinetics of Zosurabalpin (RG6006) Against *Acinetobacter baumannii*

**DOI:** 10.1093/ofid/ofad500.1733

**Published:** 2023-11-27

**Authors:** Isabelle Erbetti, Livia Ferrari, Alessia Ortombina, Paola Savoia, Antonio Felici, Caterina Bissantz, Claudia Zampaloni

**Affiliations:** Aptuit (Verona) Srl, an Evotec company, Via Fleming 4, 37135 Verona, Italy, Verona, Veneto, Italy; Aptuit (Verona) Srl, an Evotec company, Via Fleming 4, 37135 Verona, Italy, Verona, Veneto, Italy; Aptuit (Verona) Srl, an Evotec company, Via Fleming 4, 37135 Verona, Italy, Verona, Veneto, Italy; Aptuit (Verona) Srl, an Evotec company, Via Fleming 4, 37135 Verona, Italy, Verona, Veneto, Italy; Aptuit (Verona) Srl, an Evotec company, Via Fleming 4, 37135 Verona, Italy, Verona, Veneto, Italy; Roche Pharma Research and Early Development, Pharmaceutical Sciences, Roche Innovation Center Basel, F. Hoffmann-La Roche Ltd, Grenzacherstrasse 124, 4070 Basel, Switzerland, Basel, Basel-Stadt, Switzerland; F. Hoffmann-La Roche Ltd., Immunology, Infectious Disease and Ophthalmology, Roche Innovation Center Basel, F. Hoffmann-La Roche Ltd, Grenzacherstrasse 124, 4070 Basel, Switzerland, Basel, Basel-Stadt, Switzerland

## Abstract

**Background:**

Zosurabalpin (RG6006) is a novel antibiotic active against *Acinetobacter* spp., including carbapenem-resistant *Acinetobacter calcoaceticus*-*baumannii* complex organisms. Here we describe and compare the kinetics of killing in static *in vitro* and *in vivo* neutropenic murine thigh and lung infection models.

**Methods:**

The killing activity of Zosurabalpin was evaluated against 8 carbapenem resistant *A. baumannii* (CRAB) isolates (MIC range of 0.12 to 8 mg/L).

*In vitro* Time-Kill studies were performed over 24 hours at sub-MIC and multiples of the MIC (from 0.25x to 128x MIC) with regular time point samplings (from 0, 2, 4, 8, 12, 16, 20 and 24 hours), plating, and CFU counting.

The *in vivo* killing efficacy of RG6006 at 2 or 3 dose levels was assessed in 5 thigh and 5 lung infection models induced by 6 *A. baumannii* (MIC range 0.125 - 8 mg/L). Bacterial burden in matrices of interest was determined at different timepoints up to 48 hours.

**Results:**

*In vitro*, the bactericidal activity (defined as a 99.9% [or ≥ 3 log10] reduction in CFUs) of Zosurabalpin was reached in all tested isolates (lowest bactericidal concentration: 4x-32x MIC) and with a relatively slow killing kinetic (≥ 12 hours). No regrowth was observed at or above 8x/16x MIC depending on the isolate.

*In vivo*, generally, no regrowth was observed. In all 5 lung and 3 thigh infection studies, all dosing regimen achieving a net bacterial reduction after 24 hours, showed continued suppression of growth over the 48 hours period. In 2 thigh infection studies induced by isolates with MIC 4 and 8 mg/L full bactericidal efficacy was observed without any signs of regrowth at the highest dose tested.

**Conclusion:**

Zosurabalpin overall exhibited a bacterial killing effect, both *in vitro* and *in vivo*, against CRAB. The comparison of *in vitro* and *in vivo* time-kill data demonstrates that *in vitro* observed regrowth generally does not translate into, and potentially overestimates, *in vivo* regrowth.

*This project has been funded by BARDA (HHSO100201600038C)*

**Disclosures:**

**Isabelle Erbetti, n/a**, F. Hoffmann-La Roche Ltd.: Grant/Research Support **Livia Ferrari, n/a**, F. Hoffmann-La Roche Ltd.: Grant/Research Support **Alessia Ortombina, n/a**, F. Hoffmann-La Roche Ltd.: Grant/Research Support **Paola Savoia, n/a**, F. Hoffmann-La Roche Ltd.: Grant/Research Support **Antonio Felici, n/a**, F. Hoffmann-La Roche Ltd.: Grant/Research Support **Caterina Bissantz, n/a**, F. Hoffmann-La Roche Ltd.: Full time employee of Roche **Claudia Zampaloni, n/a**, F. Hoffmann-La Roche Ltd.: Full time employee of Roche

